# KL-6, a Human MUC1 Mucin, as a prognostic marker for diffuse alveolar hemorrhage syndrome

**DOI:** 10.1186/1750-1172-7-99

**Published:** 2012-12-17

**Authors:** Yoshiko Kida, Shinichiro Ohshimo, Kohei Ota, Tomoko Tamura, Tadatsugu Otani, Kazunobu Une, Takuma Sadamori, Yasumasa Iwasaki, Francesco Bonella, Noboru Hattori, Nobuyuki Hirohashi, Josune Guzman, Ulrich Costabel, Nobuoki Kohno, Koichi Tanigawa

**Affiliations:** 1 Department of Emergency and Critical Care Medicine, Graduate School of Biomedical Sciences, Hiroshima University, 1-2-3 Kasumi, Minami-ku, Hiroshima, 734-8551, Japan; 2 Department of Pneumology/Allergy, Ruhrlandklinik, University Hospital, Essen, Germany; 3 Department of Molecular and Internal Medicine, Graduate School of Biomedical Sciences, Hiroshima University, Hiroshima, Japan; 4 General and Experimental Pathology, Ruhr-University, Bochum, Germany

**Keywords:** Prognosis, Survival, Outcome, Biomarker, Interstitial lung disease, Diffuse alveolar damage

## Abstract

**Background:**

Diffuse alveolar hemorrhage syndrome is a life threatening condition with diverse etiologies. Sensitive prognostic markers for diffuse alveolar hemorrhage have not been well investigated. Serum KL-6 is a biomarker for various interstitial lung disease associated with disease activity and prognosis. The purpose of the present study was to evaluate the clinical utility of serum KL-6 level as a prognostic marker for diffuse alveolar hemorrhage.

**Methods:**

We retrospectively collected 41 consecutive patients clinically diagnosed as having diffuse alveolar hemorrhage who were admitted to the Intensive Care Unit of Hiroshima University Hospital between 2004 and 2011. Correlation between prognosis and age, sex, laboratory findings including serum KL-6, radiological findings, ventilatory modes or therapeutic regimens were evaluated.

**Results:**

Baseline and peak serum KL-6 levels were significantly higher in non-survivors compared with survivors. An increase in KL-6 levels during the initial week was associated with a subsequent deterioration of the oxygenation index. Higher baseline KL-6 levels and higher peak KL-6 levels were strongly correlated with death. With a cut-off level of 700 U/mL for peak KL-6, the sensitivity, specificity and accuracy for non-survival were 75%, 85% and 78%, respectively. In the multivariate analysis, only the peak KL-6 level ≥700 U/ml was an independent poor prognostic factor for diffuse alveolar hemorrhage.

**Conclusions:**

Peak serum KL-6 level ≥700 U/ml may become a clinically useful marker of poor prognosis for diffuse alveolar hemorrhage.

## Background

Diffuse alveolar hemorrhage (DAH) syndrome is a life threatening condition associated with diverse etiologies including infection, excessive anticoagulation and vasculitis [1]. Repeated episodes of DAH may lead to irreversible pulmonary fibrosis [1] and progressive obstructive pulmonary dysfunction [2]. Low PaO_2_/F_I_O_2_ (P/F) ratio, high multi-organ dysfunction score and non-autoimmune etiology have been reported to be poor prognostic factors for DAH [3]. However, reliable prognostic markers of DAH have not been well investigated.

KL-6, a complex sialo-carbohydrate glycoprotein present in the human MUC1 mucin, is expressed on the surface of type II pneumocytes in the alveolar space [4]. KL-6 is a sensitive serum marker for various interstitial lung diseases including idiopathic pulmonary fibrosis, radiation pneumonitis, drug-induced pneumonitis, collagen vascular disease-associated interstitial pneumonitis, extrinsic allergic alveolitis, pulmonary sarcoidosis, pulmonary alveolar proteinosis and cystic fibrosis [5-7]. Serial changes of serum KL-6 were useful to predict the short-term prognosis in rapidly progressive idiopathic pulmonary fibrosis [8], and baseline serum KL-6 levels were related to the long-term survival in idiopathic pulmonary fibrosis [9]. Serum KL-6 levels are therefore likely to be of use for evaluating the prognosis of DAH.

The purpose of the present study was to evaluate the clinical utility of serum KL-6 level as a prognostic marker for DAH.

## Methods

### Study subjects

Consecutive patients who were admitted to the ICU and diagnosed as DAH between 2004 and 2011 were retrospectively studied. Patients’ characteristics including age, sex, laboratory findings, radiological findings, ventilatory modes or therapeutic regimens were extracted from the medical records. The vital status of the patients was determined by reviewing medical records, death certificates, as well as by phone interviews.

Serum KL-6 and lactate dehydrogenase (LDH), prothrombin time-international normalized ratio (PT-INR), activated partial thromboplastin time, D-dimer and hemoglobin levels and platelet counts were included as variables for analysis when taking routine blood tests and performing BAL.

Baseline KL-6 was defined as serum KL-6 level at admission, and peak KL-6 was defined as the highest serum KL-6 level during the follow-up. KL-6 was measured once-weekly. The etiologies of DAH were grouped into 5 categories: pulmonary infection, excessive anticoagulation, vasculitis, interstitial pneumonia or idiopathic based on the different pathogenesis in our patients. The diagnosis of vasculitis was defined by histological evidence, high titers of anti-neutrophil cytoplasmic antibodies, anti- deoxyribonucleic acid antibodies or anti-basement membrane antibodies [10,11]. Correlations between survival and clinical variables were evaluated.

### Definition of DAH

Clinical diagnosis of DAH was based on the findings of diffuse ground glass or airspace-filling opacities on chest radiograph and computed tomography, and BAL fluid showing progressively bloodier returns and the presence of 20% or more hemosiderin-laden macrophages [12]. Exclusion criteria were apparent deterioration of left heart failure and contact bleeding with bronchoscope.

### BAL

The procedure of BAL was done as previously described [13]. In brief, a flexible bronchoscope was wedged into a segmental bronchus of the middle lobe or the lingula. Sterile isotonic saline at 37°C was instilled in three 50 ml aliquots up to a total volume of 150 ml, with immediate aspiration by gentle suction after each aliquot. The total of recovered BAL fluid fractions were mixed and immediately filtered through two layers of gauze, and centrifuged at 500 g for 10 min at room temperature. Slides were stained with Prussian blue to detect haemosiderin-laden macrophages [12]. After incubation of the slides with 1% hydrochloric acid and 2% potassium ferricyanide (Katayama Medical Co. Ltd., Osaka, Japan) for 20 minutes, slides were counterstained with Kernechtrot stain (Tokyo Chemical Industry Co. Ltd., Tokyo, Japan). A total of 200 or more macrophages were examined for calculating a percentage of Prussian blue positive cells. The BAL fluid samples also underwent routine microbiologic testing for detecting bacteria and viruses.

### Statistical analysis

Data are expressed as mean ± standard deviation. Comparison of non-normally distributed variables between groups was done with the Mann-Whitney’s *U* test or Fisher’s PLSD test. Analysis of changes in non-normally distributed variables between groups was done with the Wilcoxon’s rank test or repeated measures of analysis of variance. Comparison of categorical variables between two groups was done with the χ^2^ test. The probability of survival was estimated with the Kaplan-Meier method, and the differences in survival rates were evaluated by log-rank test. Multivariate analysis of prognostic factors was done using the Cox regression model. All statistical analyses were done using SPSS version 13.0 for Windows (SPSS Inc., Chicago, IL). Differences were considered statistically significant when the p value was <0.05.

## Results and discussion

### Patient characteristics

There were 25 males and 16 females with a median age of 69 (range, 16–83) years. There were 27 never smokers, 12 former smokers, 2 current smokers, respectively. The etiologies of DAH were pulmonary infection (n=19), excessive anticoagulation (n=9), vasculitis (n=6), interstitial pneumonia (n=2) and idiopathic (n=5). Thirteen patients survived, and 28 died. The ICU mortality was 54% (22/41), the in-hospital mortality was 68% (28/41), and the 28-day mortality was 32% (13/41), respectively. No significant differences were observed in age, sex, smoking status, radiological findings, use of mechanical ventilation, baseline P/F ratio, and laboratory markers at admission between the groups (Table 1). Use of methylprednisolone pulse after admission was more frequent, prothrombin time-international normalized ratio and activated partial thromboplastin time were higher, and duration of ICU stay was significantly longer in non-survivors than survivors (p=0.038, 0.043, 0.029, 0.005, respectively). Meanwhile, P/F ratio 48 hrs after admission was significantly lower in non-survivors than survivors (p=0.023). There was no difference in the frequency of etiologies of DAH between survivors and non-survivors.

**Table 1:** Differences in clinical characteristics between survivors and non-survivors with DAH

**Variables**	**Survivors, n(%)**	**Non-survivors, n(%)**	**p-value**
	**(n=13)**	**(n=28)**	
Age ≥70(yrs)	3 (23)	16 (57)	0.052*
Male sex	8 (62)	17 (61)	>0.99*
Never smoker	7 (54)	19 (70)	0.48*
Bilateral pulmonary involvement	9 (69)	24 (86)	0.24*
Consolidation on chest x-ray	7 (54)	15 (54)	>0.99*
GGA on chest x-ray	9 (69)	22 (79)	0.70*
Use of mechanical ventilation	10 (77)	26 (93)	0.30*
Mode of mechanical ventilation			0.28*
SIMV	5 (12)	9 (22)	
APRV	3 (7)	8 (20)	
HFOV	0 (0)	6 (15)	
CPAP/ PSV	2 (5)	1 (2)	
O_2_ mask/ NIPPV	3 (7)	4 (10)	
Preceding interstitial pneumonia	0 (0)	3 (11)	0.54*
Treatment			
Methylprednisolone pulse	5 (38)	21 (75)	0.038*
Immunosupressant	2 (15)	5 (18)	>0.99*
Plasema exchange	1 (8)	4 (14)	>0.99*
Baseline PaO_2_/F_I_O_2_ ratio	207±80	185±70	0.47^#^
P/F ratio 48 hrs after admission	314±86	250±114	0.023^#^
Laboratory markers at admission			
PT-INR	1.2±0.3	1.8±1.4	0.043^#^
APTT (sec)	29.6±4.7	42.0±18.0	0.029^#^
D-dimer	17.4±29.0	18.8±20.5	0.46^#^
Platelet (x10^3^/mL)	185±88	142±103	0.09^#^
Hemoglobin (g/mL)	10.1±2.3	9.3±2.3	0.26^#^
LDH (IU/L)	567±399	736±1045	0.88^#^
Duration of ICU stay (days)	15±15	28±20	0.005^#^

^*^Fisher’s exact test; ^#^ Mann-Whitney’s *U* test.

GGA, ground glass attenuation; SIMV, synchronized intermittent mandatory ventilation.

APRV, airway pressure release ventilation; HFOV, high-frequency oscillatory ventilation.

CPAP, continuous positive airway pressure; PSV, pressure support ventilation.

NIPPV, non-invasive positive pressure ventilation.

PT-INR, prothrombin time-international normalized ration.

APTT, acivated partial thromboplastin time; LDH, lactate dehydrogenase.

ICU, intensive care unit.

### Baseline and peak serum KL-6 levels

**Figure 1:**
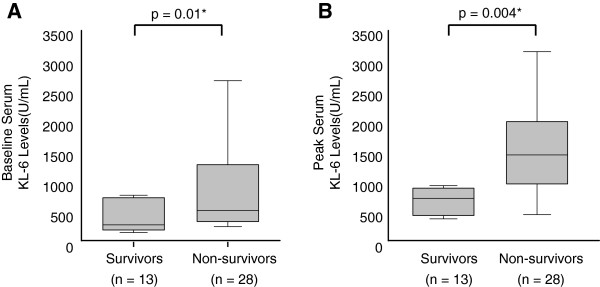
**Box plot graphs showing the range of serum KL-6 levels in diffuse alveolar hemorrhage syndrome.** (**A**) baseline and (**B**) peak serum KL-6 levels in survivors and non-survivors.

The baseline and peak serum KL-6 levels in 41 patients are shown in Figure 1. The median duration before serum KL-6 level reached the peak level was 11±10 days. The median duration from the time of peak serum KL-6 level to death was 23±23 days. Baseline and peak serum KL-6 levels were significantly higher in non-survivors than in survivors (baseline levels, 845±831 vs 492±606 U/mL, p=0.01; peak levels, 1471±1648 vs 659±813 U/mL, p=0.004, respectively).

### Correlation between serum KL-6 levels and the use of mechanical ventilation

**Figure 2:**
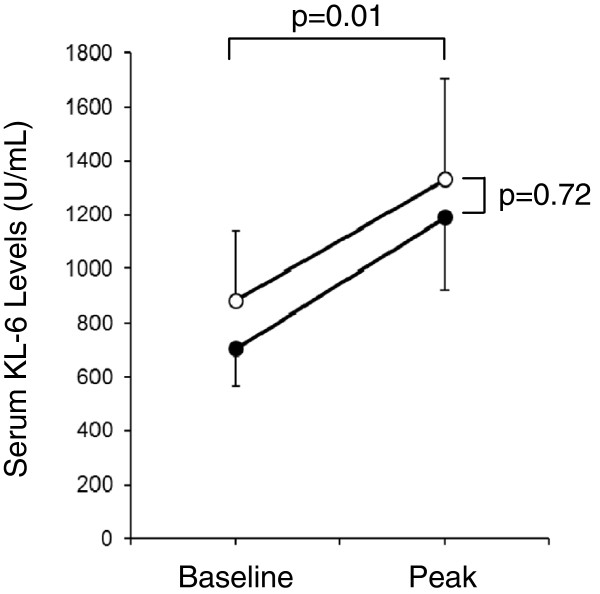
**Baseline and peak serum KL-6 levels in patients treated with mechanical ventilation (n=34, closed circles) and without mechanical ventilation (n=7, open circles).** Data are expressed as mean ± standard error of mean. Mean serum KL-6 levels increased in both groups (p=0.01). There was no difference in serum KL-6 levels according to the use of mechanical ventilation (p=0.72).

In our cohort, 34 patients were intubated, whereas the remaining 7 patients were treated without mechanical ventilation. Mean serum KL-6 levels increased during the follow-up in both groups (p=0.01; Ventilated patients, baseline 703±799 U/mL, peak 1,189±1,567 U/mL; Non-ventilated patients, baseline 881±694 U/mL, peak 1,330±995 U/mL; Figure 2). There was no difference in serum KL-6 levels according to the use of mechanical ventilation (p=0.72).

### Serial changes in serum KL-6 in association with P/F ratio or oxygenation index

**Figure 3:**
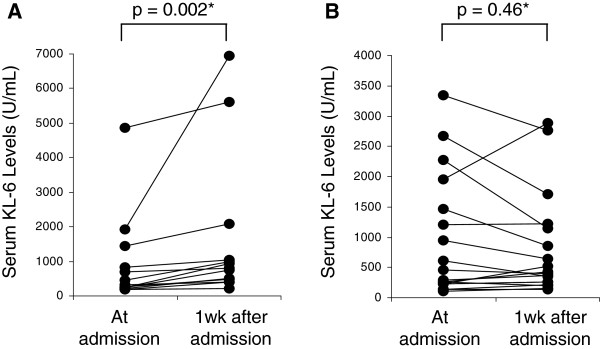
**Serial changes in serum KL-6 levels at admission and one week after admission.** (**A**) Patients who showed a decrease in PaO_2_/F_I_O_2_ ratio during the initial week (n=13). (**B**) Patients who showed an increase in oxygenation index during the initial week (n=16).

Serial changes in serum KL-6 in patients with DAH are shown in Figure 3. Patients who showed a decrease in P/F ratio (n=13, Figure 3A) or an increase in oxygenation index (n=16, Figure 3B) during the initial week showed a significant increase in serum KL-6 levels (p=0.002, p=0.003, respectively). In Figure 3A, two subjects appear to have extremely high levels of KL-6 one week after admission. When they were excluded to avoid statistical error, the increase in serum KL-6 levels during the initial week was still significant (p=0.003).

### Receiver operating characteristic curve analysis

**Figure 4:**
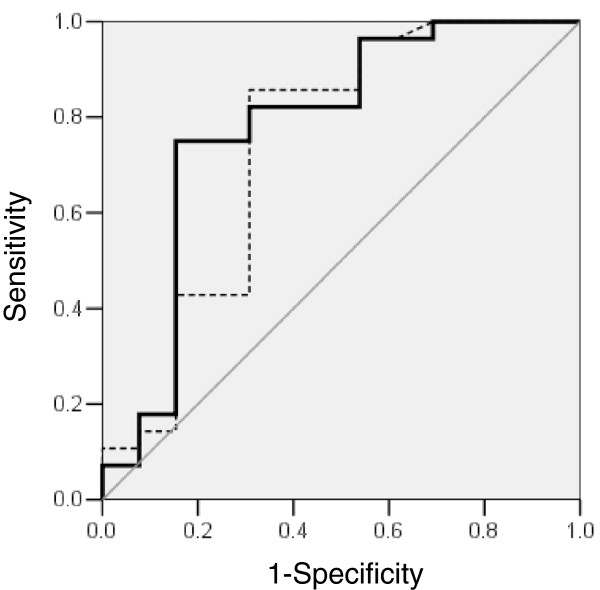
**Receiver operating characteristic curve analysis.** The curves show the diagnostic power of baseline and peak serum KL-6 levels for the detection of non-survival in diffuse alveolar hemorrhage syndrome. Narrow dotted line indicates baseline serum KL-6 levels; heavy continuous line indicates peak serum KL-6 levels.

Receiver operating characteristic curve analysis was used to evaluate the sensitivity, specificity and accuracy of baseline and peak serum KL-6 levels in relation to non-survival (Figure 4). The larger area under the curve which is associated with non-survival was found for peak KL-6 with 0.78 (95% confidence interval (CI), 0.61 to 0.96) compared with baseline KL-6 with 0.74 (95% CI, 0.56 to 0.93). When the cut-off levels were set at the closest point to 100% sensitivity and 100% specificity, the levels in the prediction of non-survival were 700 U/mL for peak KL-6 (sensitivity, 75%; specificity, 85%; accuracy, 78%, respectively) and 240 U/mL for baseline KL-6 (sensitivity, 86%; specificity, 69%; accuracy, 81%, respectively).

### Correlation between serum KL-6 levels and overall survival

**Figure 5:**
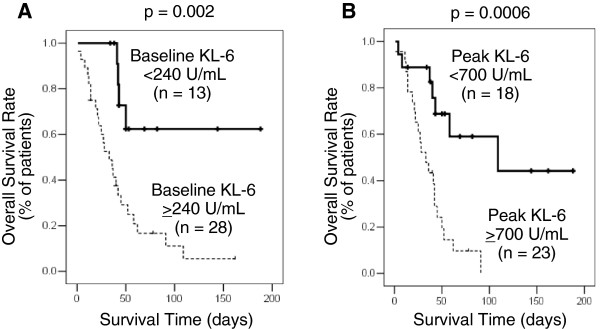
**Kaplan-Meier analysis.** The graph shows overall survival in patients with diffuse alveolar hemorrhage syndrome in relation to their (**A**) baseline and (**B**) peak serum KL-6 levels.

The Kaplan-Meier analysis showed that higher baseline KL-6 levels were associated with a shorter median survival period (p=0.002) (Figure 5A). The same was true for higher peak KL-6 levels (p=0.0006) (Figure 5B).

In the univariate survival analysis, P/F ratios <200 48 hours after admission (Hazard ratio, 2.58, 95% CI, 1.17-5.68; p=0.018), baseline serum KL-6 levels ≥240 U/mL (Hazard ratio, 4.72, 95% CI, 1.63-13.7; p=0.004) and peak serum KL-6 levels ≥700 U/mL (Hazard ratio, 4.43, 95% CI, 1.76-11.1; p=0.002) were associated with non-survival, whereas no correlations were found between survival and age, sex, smoking status, radiological findings, baseline P/F ratio prothrombin time-international normalized ratio levels and activated partial thromboplastin time (Table 2). In the multivariate survival analysis only peak serum KL-6 levels ≥700 U/mL were associated with non-survival (Hazard ratio, 3.95, 95% CI, 1.14-13.7; p=0.031), after adjustment for age, sex, smoking status, extent of pulmonary involvement and P/F ratio 48 hrs after admission.

**Table 2:** Cox’s proportional analysis of the overall survival

**Variable**	**Unfavorable/Favorable**	**β**	**HR (95%CI)**	p-value^*^
Univariate analysis				
Age(yrs)	<70/ ≥70	0.34	1.40 (0.66-2.97)	0.38
Sex	female/ male	0.18	1.99 (0.56-2.58)	0.64
Smoking status	never/ smoker	0.43	1.54 (0.67-3.55)	0.31
Pulmonary involvement	bilateral/ unilateral	0.70	2.02 (0.68-6.02)	0.21
Baseline P/F ratio	<200/ ≥200	0.47	1.60 (0.74-3.45)	0.23
P/F ratio 48 hrs after admission	<200/ ≥200	0.95	2.58 (1.17-5.68)	0.018
PT-INR	≥2/ <2	0.10	1.11 (0.38-3.23)	0.85
APTT (sec)	≥30/ <30	0.28	1.33 (0.56-3.17)	0.52
Baseline KL-6 level (U/mL)	≥240/ <240	1.55	4.72 (1.63-13.7)	0.004
Maximum KL-6 level (U/mL)	≥700/ <700	1.49	4.43 (1.76-11.1)	0.002
Multivariate analysis *				
Baseline KL-6 level (U/mL)	≥240/ <240	0.99	2.70 (0.75-9.67)	0.13
Maximum KL-6 level (U/mL)	≥700/ <700	1.37	3.95 (1.14-13.7)	0.031

HR, hazard ratio; CI, confidence interval; GGA, ground glass attenuation; P/F, PaO_2_/F_I_O_2_.

PT-INR, prothrombin time-international normalized ratio.

APTT, activated partial thromboplastin time.

* Adjusted for age, sex, smoking status, extent of pulmonary involvement and P/F ratio 48 hrs after admission.

## Discussion

This study showed that baseline and peak serum KL-6 levels are significantly higher in non-survivors compared with survivors of DAH. The increase in serum KL-6 levels during the initial week was associated with a subsequent deterioration in P/F ratio or oxygenation index. With a cut-off level of 700 U/mL for peak serum KL-6, the sensitivity, specificity and accuracy for non-survival were 75%, 85% and 78%, respectively. In the multivariate analysis, peak serum KL-6 ≥700 U/mL was a significant prognostic factor for poor outcome in DAH.

Previous studies have shown several factors associated with mortality in DAH. Holguin *et al*. demonstrated that mechanical ventilation, admission to ICU and blood transfusion were associated with increased mortality within 28 days after admission in patients with ANCA-related pulmonary vasculitis [14]. Afessa *et al*. demonstrated in a retrospective study of 48 hematopoietic stem cell transplant recipients that autologous transplant and early-onset DAH were associated with good prognosis [15]. To date, no single serum marker has been shown to be related to the prognosis of DAH, and most of the previous studies focused on immunologically mediated DAH such as vasculitis [16]. The patient population in our study, however, included severe pulmonary infection and excessive anticoagulation as major etiologies of DAH, and this population has not been well investigated.

The mechanisms of the increase in serum KL-6 levels in various fibrotic lung diseases are thought to be due to an overexpression of KL-6 by regenerating alveolar type II pneumocytes, and/or due to an increased permeability following disintegration of the alveolar-vessel barrier [17,18]. The alveolar epithelial damage and the following excessive fibroblast accumulation in DAH appear to be associated with the increment in serum KL-6. As shown in Figure 1, 2 and 3, the range of both baseline and peak serum KL-6 levels are wide, indicating the individual variation. This variation might be attributed to the delay of first admission after the onset of DAH. Although both baseline and peak serum KL-6 levels were prognostic markers for poor outcome in the survival analysis, peak serum KL-6 levels had a stronger predictive value, suggesting that the advanced alveolar epithelial damage is likely the most important factor for death in DAH.

Mechanical ventilation itself may potentially cause acute alveolar damage and a resultant DAH. Previous studies have demonstrated that plasma level of KL-6 was increased with injurious ventilator settings, and may serve as a biomarker of ventilator-associated lung injury (VALI) [19]. All patients intubated in our study were treated with lung-protective ventilatory modalities to avoid VALI as much as possible, and no significant differences were observed in the use and modes of mechanical ventilation (Table 1). As a result, no significant difference was observed in serum KL-6 levels according to the use of mechanical ventilation (Figure 2).

A recent study has clarified that the epitope of KL-6 monoclonal antibody involved sulfate and sialic acid residues, which may be regulated by Gal6ST gene [20]. We have previously shown that the purified KL-6 molecule has chemotactic, proliferative and anti-apoptotic effects on fibroblasts *in vitro*, and that the proliferative and anti-apoptotic effects of KL-6 are additive to those of transforming growth factor-β [21,22].The increment of KL-6 in the alveolar space may be initially a resultant epiphenomenon of epithelial damage in DAH. Subsequently, however, through specific receptors, KL-6 may be able to accelerate epithelial damage and lead to fibrosis in DAH. Further *in vitro* studies are necessary to support the role of KL-6 in the development of DAH.

A potential limitation of our study is its retrospective design and the relatively limited number of patients enrolled. Because of the lack of separate disease controls such as acute exacerbation of idiopathic pulmonary fibrosis in our study, the specificity of KL-6 as a prognostic marker in DAH has not been shown. KL-6 is likely to increase in various types of lung injury resulting in epithelial damage, and may not be unique for DAH. Because the concentrations of KL-6 in the blood and BAL fluid were not simultaneously evaluated in our study, the interaction of systemic and local changes in DAH could not be investigated. Another potential limitation is the selection bias of the patients. The enrolled patients presented heterogeneous etiologies resulting in DAH. In addition, the enrolled patients had severe DAH, because we exclusively studied patients who were referred to the ICU unit. Therefore, our result may not be valid for patients with the milder forms of DAH which are clinically more common.

In conclusion, peak serum KL-6 level ≥700 U/ml may become a clinically useful marker of poor prognosis for DAH. Further longitudinal studies with a larger number of patients are required to support our findings.

### Ethical standards

The experiments in this study comply with the current laws of Japan and Germany.

## Abbreviations

BAL: Bronchoalveolar lavage; CI: Confidence interval; DAH: Diffuse alveolar hemorrhage; ICU: Intensive care unit; P/F: PaO_2_/F_I_O_2_ ; VALI: Ventilator-associated lung injury.

## Competing interests

Kohno has a royalty income concerning the discovery and the clinical application of KL-6. However, he has no significant conflicts of interest on the theme discussed in this article. Other authors have no financial support. No significant conflicts of interest exist with any companies/organizations whose products or services may be discussed in this article.

## Authors’ contributions

YK carried out the patient collection, made the database, carried out the statistical analyses and drafted the manuscript. SO conceived of the study, carried out the statistical analyses and helped to draft the manuscript. KO, TT, TO, KU and TS carried out the patient treatment and collected the data. YI, NH, NH, NK and KT participated in the design of the study and coordination, and helped to draft the manuscript. FB, JG and UC participated in the design of the study, carried out the statistical analyses and helped to draft the manuscript. All authors read and approved the final manuscript.
